# The soluble form of pan-RTK inhibitor and tumor suppressor LRIG1 mediates downregulation of AXL through direct protein–protein interaction in glioblastoma

**DOI:** 10.1093/noajnl/vdz024

**Published:** 2019-09-06

**Authors:** Virginie Neirinckx, Ann-Christin Hau, Anne Schuster, Sabrina Fritah, Katja Tiemann, Eliane Klein, Petr V Nazarov, André Matagne, Martyna Szpakowska, Max Meyrath, Andy Chevigné, Mirko H H Schmidt, Simone P Niclou

**Affiliations:** 1 NorLux Neuro-Oncology Laboratory, Department of Oncology, Luxembourg Institute of Health, Luxembourg; 2 Proteome and Genome Research Unit, Department of Oncology, Luxembourg Institute of Health, Luxembourg; 3 Center for Protein Engineering, University of Liège, Liège, Belgium; 4 Immuno-Pharmacology and Interactomics, Department of Infection and Immunity, Luxembourg Institute of Health, Luxembourg, Germany; 5 Molecular Signal Transduction Laboratories, Institute for Microscopic Anatomy and Neurobiology, University Medical Center of the Johannes Gutenberg University, Mainz, Germany; 6 German Cancer Consortium (DKTK), Heidelberg, Germany; 7 German Cancer Research Center (DKFZ), Heidelberg, Germany

**Keywords:** AXL, EGFR, glioblastoma, LRIG1, receptor tyrosine kinase

## Abstract

**Background:**

Targeted approaches for inhibiting epidermal growth factor receptor (EGFR) and other receptor tyrosine kinases (RTKs) in glioblastoma (GBM) have led to therapeutic resistance and little clinical benefit, raising the need for the development of alternative strategies. Endogenous LRIG1 (Leucine-rich Repeats and ImmunoGlobulin-like domains protein 1) is an RTK inhibitory protein required for stem cell maintenance, and we previously demonstrated the soluble ectodomain of LRIG1 (sLRIG1) to potently inhibit GBM growth in vitro and in vivo.

**Methods:**

Here, we generated a recombinant protein of the ectodomain of LRIG1 (sLRIG1) and determined its activity in various cellular GBM models including patient-derived stem-like cells and patient organoids. We used proliferation, adhesion, and invasion assays, and performed gene and protein expression studies. Proximity ligation assay and NanoBiT complementation technology were applied to assess protein–protein interactions.

**Results:**

We show that recombinant sLRIG1 downregulates EGFRvIII but not EGFR, and reduces proliferation in GBM cells, irrespective of their EGFR expression status. We find that sLRIG1 targets and downregulates a wide range of RTKs, including AXL, and alters GBM cell adhesion. Mechanistically, we demonstrate that LRIG1 interferes with AXL but not with EGFR dimerization.

**Conclusions:**

These results identify AXL as a novel sLRIG1 target and show that LRIG1-mediated RTK downregulation depends on direct protein interaction. The *pan*-RTK inhibitory activity of sLRIG1 warrants further investigation for new GBM treatment approaches.

**Key points**

1. Soluble LRIG1 alters GBM cell proliferation, survival, adhesion, and invasion.
2. Soluble LRIG1 simultaneously targets multiple RTKs, including AXL.
3. LRIG1 directly interacts with AXL, which is required for receptor downregulation.

**Importance of the Study**

Endogenous RTK inhibitors are poorly studied and have so far not been exploited in a therapeutic perspective for cancer patients. We describe for the first time the antitumor activity of a recombinant RTK inhibitory protein (LRIG1) that strongly alters cell proliferation and adhesion, which provides the basis for a novel protein-based therapeutic concept targeting multiple RTKs. We confirm that soluble LRIG1 downregulates multiple RTKs, and identifiy AXL as a novel LRIG1 target, known as a driver of GBM progression and resistance to anti-EGFR therapy. The finding that downregulation of AXL is dependent on direct protein–protein interaction provides novel insight into the mechanism of LRIG1-induced RTK regulation. Our data indicate that recombinant LRIG1 is endowed with *pan-*RTK activity and has strong potential for overcoming therapeutic resistance against small molecule inhibitors in GBM.

Aberrant growth factor signaling through receptor tyrosine kinases (RTKs) is a hallmark of cancer, and triggers abnormal cell proliferation, enhanced motility, and therapeutic resistance of many solid tumors including glioblastoma (GBM).^1^ The Cancer Genome Atlas (TCGA) project confirmed alterations in RTK genes or downstream pathways as essential drivers of GBM, detectable in >80% of patients.^2^ In particular, the epidermal growth factor receptor (EGFR) is predominantly amplified/mutated. The most common structural variant in GBM, EGFR variant III (EGFRvIII), is characterized by a truncated extracellular domain lacking the ligand-binding site and is constitutively active and highly oncogenic.^3^ Altered signaling via EGFR and/or EGFRvIII is involved in GBM proliferation and invasion.^4^ At the molecular level, EGFR and EGFRvIII interplay^5^ or interact with other RTKs^6,7^ to fine-tune these oncogenic processes.

Anti-EGFR therapies consequently have been in the focus of attention: antibodies, tyrosine kinase inhibitors (TKIs), or vaccines were tested in GBM patients, but largely remained unsuccessful,^8–10^ due to limited drug delivery to the brain, tumor heterogeneity, and acquired resistance. Resistance to EGFR targeting was associated with activation of other RTKs such as EGFRvIII,^11^ InsR/IGF1R,^12^ or PDGFRβ.^13^ In addition, RTKs converge on common signaling pathways, which further hinders their specific inhibition by targeted drugs.

LRIG1 (Leucine-rich Repeats and ImmunoGlobulin-like domains 1) acts as a negative regulator of numerous RTKs, and modulates proliferation, invasion, and angiogenesis.^14^ LRIG1 was identified as a *bonafide* tumor suppressor^15,16^ and stem cell marker,^17,18^ further associated with good prognosis in several cancers.^19^ Although the mechanism of action remains a matter of debate, this membrane protein is thought to downregulate RTK signaling through receptor ubiquitination, internalization, and degradation.^14^ The extracellular part of LRIG1 can be shed from the membrane and released in the extracellular space as soluble LRIG1 (sLRIG1) and is sufficient to inhibit EGFR signaling.^20,21^ We previously reported that the treatment of GBM patient-derived orthotopic xenografts (PDOX) with sLRIG1 reduced tumor growth and improved mouse survival, in GBM with or without EGFR amplification. sLRIG1 also impaired proliferation of U87-derived cells with different EGFR expression status. These data suggested that sLRIG1 reduced GBM progression at least partially through EGFR-independent mechanisms, potentially targeting other mediators of tumor growth.^22^

Here, we have engineered, produced, and purified a recombinant human sLRIG1 protein (rh-sLRIG1) that reduces proliferation of GBM cells and patient-derived organoids in vitro and affects cell adhesion. sLRIG1 downregulates EGFRvIII, but not wild-type EGFR. We show that sLRIG1 affects multiple RTKs at once, and we identify AXL as a novel target. Mechanistically, sLRIG1 hinders AXL dimerization, but does not interfere with EGFR.

## Materials and Methods

### Cell Lines and Primary Patient-Derived GBM Organoids

Glioblastoma stem-like cells (GSCs) and U87-derived cell lines were cultured as described previously.^22,23^ Collection and use of patient tumor tissue samples was performed after patients provided informed consent, and approved by the appropriate local ethics committee (National Ethics Committee for Research [CNER], Luxembourg, REC-LRNO-20110708). Establishment of orthotopic patient-derived xenografts (P3, T16, and T188) and animal care were described previously.^22,23^ Surgical procedures were performed in accordance with the regulations of the European Directive on animal experimentation (2010/63/EU). At tumor endpoint, brain tissue was harvested and processed with Neural Tissue Dissociation kit (Miltenyi) followed by Mouse Cell Depletion kit (Miltenyi). One thousand single cells were seeded per well (384-well plate) for tumor organoid formation.

### Production of Recombinant Human sLRIG1 (rh-sLRIG1) and Control IgG, and Cell Treatment

The LRIG1 ectodomain (A35-S779) was C-terminally tagged with a Histidine tag, shuttled in a baculovirus vector (Gateway) and expressed in Sf9 cells. The recombinant protein was purified using an Imac column on an Äkta purifier (GE Healthcare), eluted using an imidazole gradient, and dialyzed against phosphate-buffered saline (PBS). For cell-based assays, rh-sLRIG1 protein or IgG control protein was added to the culture medium at a concentration of 15 µg/mL (= 0.18 µM), and incubated for 6 days.

### Circular Dichroism and Fluorescence Spectroscopy

Far-UV circular dichroism (CD) spectra (195–260 nm) were recorded with a Jasco J-810 spectropolarimeter, with a protein concentration of 0.1 mg/mL (1.15 µM). Data are presented as the molar residue ellipticity ([Ɵ]_MRW_). Intrinsic fluorescence spectrum was recorded with a Cary Eclipse spectrofluorimeter (Varian), with a protein concentration of 0.01 mg/mL. The excitation wavelength was 280 nm and emission was recorded in the 300–440 nm range, at a rate of 600 nm·min^−1^ (Supplementary Methods).

### Cell Proliferation Assay

Proliferation rates were obtained by plating 1 × 10^5^ cells per well in 6-well plates. After 6 days, cells were counted using an automated cell counter (Countess, Life Technologies), and the cell number fold change was calculated.

### Viability and Cytotoxicity Assays

PDOX-derived cells (P3, T16, and T188) were freshly isolated and seeded in 384-well plates. After spheroid formation, we applied rh-sLRIG1 or R428 (S2841, SelleckChem) and performed CellTiter-Glo2.0 and CellTox-Green assays (Promega), according to the manufacturer’s instructions. IgG and DMSO were used as negative controls. Analysis and IC50 determination were performed via GraphPad Prism 7 software (Supplementary Methods).

### Invasion Assay

Boyden chamber assays were performed using inserts coated with collagen type I (Sigma) and ECM proteins (Sigma). After 16 hours, invaded cells were fixed with 4% paraformaldehyde and stained with Crystal Violet. Cells were counted in five representative fields/insert, and countings were corrected for proliferation to obtain the percentage of invasion (Supplementary Methods).

### Gene Expression Analysis

Total RNA of U87-EGFRvIII and U87-EGFRvIII-sLRIG1 cells was extracted using the RNeasy Mini Kit (Qiagen). Total RNA was hybridized on Human Gene 2.0 ST Arrays (Affymetrix) in accordance with the manufacturer’s instructions. A list of differentially expressed genes (DEG) was created by analysis of variance (ANOVA) with FDR < 0.01 and an absolute fold change FC > 2. Raw data are accessible on GEO (E-MTAB-7474; Supplementary Methods).

### Reverse Transcription and qRT–PCR

cDNA was synthesized using iScript Reverse Transcriptase (BioRad) and applied for real-time PCR reaction in a Via7 instrument using Fast SYBR Green (Applied Biosystems) and specific primers (Eurogentec; Supplementary Table S1; Supplementary Methods).

### Western Blot and Antibody Arrays

Protein extracts were resolved in NuPage 4–12% BisTris gels (ThermoFisher), and blotted onto a PVDF membrane according to standard protocols. Blots were probed with primary antibodies at 4°C overnight. Secondary antibodies (Jackson ImmunoResearch) were applied, and blots were developed with a chemiluminescent substrate (ThermoFisher). Human phospho-RTK antibody array (ARY001B, R&D Systems) was performed following the manufacturer’s instructions (Supplementary Methods).

### Immunofluorescence

GBM cells were fixed with 4% paraformaldehyde and blocked for 1 hour with PBS supplemented with 0.1% Triton X-100 (PBS-T) and 10% FBS. Primary antibodies were diluted in PBS-T and incubated for 2 hours at room temperature. After PBS washing, cells were incubated with Alexa Fluor 488-, 555-, or 647-conjugated antibodies (ThermoFisher). Image acquisition and analysis were performed using a LSM880 Confocal microscope and ZEN2 software (Zeiss;Supplementary Methods).

### In Situ Proximity Ligation Assay

Proximity ligation assay (PLA) was performed to detect interaction between sLRIG1 and AXL. Cells were incubated overnight with primary antibodies, and DuoLink detection probes (Sigma) were used according to the manufacturer’s instructions. Image acquisition was performed using Ni-E microscope (Nikon) and analysis was done with Image J software (Supplementary Methods).

### NanoBiT Complementation Assay

RTK dimerization and interaction with LRIG1 were monitored by NanoLuc complementation assay (NanoBiT, Promega).^24–26^ U87 cells were transfected with pNBe vectors containing human EGFR, AXL, or LRIG1, C-terminally fused to LgBiT or SmBiT. For competition experiments, pIRES plasmids containing untagged EGFR, AXL, LRIG1, or sLRIG1 were co-transfected. Forty-eight hours posttransfection, cells were harvested and distributed into white 96-well plates, incubated with ligands of interest, and then with Nano-Glo Live Cell substrate. RTK dimerization or interaction with LRIG1 were evaluated with a ClarioStar luminometer (BMG LabTech). The signal is reported as a ratio to “untreated” control condition (without ligand), being set to 1 (Supplementary Methods).

### Statistical Analysis

Data were analyzed using the GraphPad Prism 7 software. Results are reported as mean ± standard error of the mean, with the *n* described as the number of biological replicates. Data were submitted to Student *t* tests or ANOVA (two-tailed), and statistical significance was set at *P* < .05.

## Results

### Recombinant Soluble LRIG1 Downregulates EGFRvIII, and Reduces Proliferation of GBM Cells and Patient-Derived Organoids, Independent of Their EGFR Status

**Figure 1.**
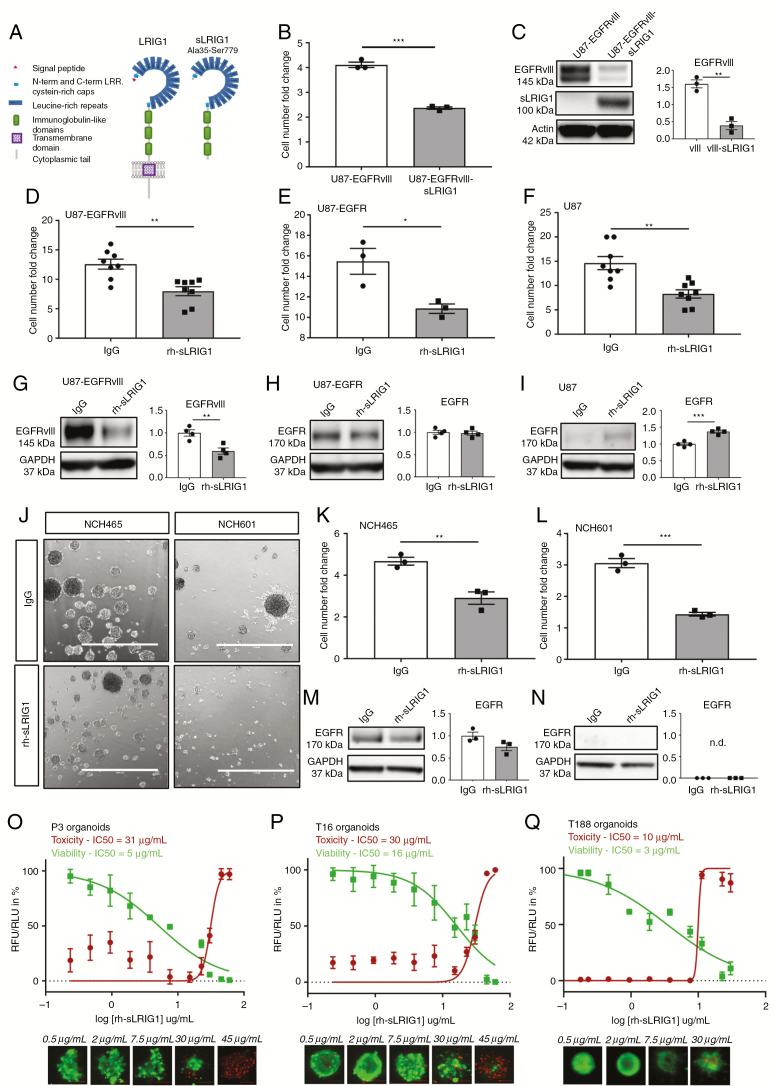
Soluble LRIG1 downregulates EGFRvIII but not wild-type EGFR, and reduces proliferation in GBM cells and patient-derived organoids. (A) Schematic representation of full-length LRIG1 and soluble LRIG1 (sLRIG1). sLRIG1 includes LRRs and Ig domains (A35-S779). (B) Overexpression of sLRIG1 in U87-EGFRvIII reduced proliferation (*n* = 3; *P* = .0001), and (C) downregulated EGFRvIII protein levels (*n* = 3; *P* = .0018). (D–F) Recombinant human soluble LRIG1 (rh-sLRIG1) decreased cell proliferation (cell number fold change to day of seeding) in U87-EGFRvIII (*n* = 8; *P* = .0028), U87-EGFR (*n* = 3; *P* = .026) and U87 cells (*n* = 8; *P* = .0014) after 6 d of incubation. (G–I) rh-sLRIG1 decreased EGFRvIII protein levels in U87-EGFRvIII (*n* = 4; *P* = .0066), but not EGFR. (J) Treatment of GSCs with rh-sLRIG1 reduced sphere size and number (scale bar = 1 mm), resulting in a (K–L) decreased proliferation in NCH465 (*n* = 3; *P* = .007) and NCH601 (*n* = 3; *P* = .0005). (M–N) EGFR protein levels were unchanged in NCH465 (*n* = 3; *P* = 0.112) and not detected in NCH601. (O) rh-sLRIG1 decreases P3 viability in a concentration-dependent fashion (IC50_Viab_ = 5 µg/mL), but has a cytotoxic effect only at high concentrations (IC50_Tox_ = 31 µg/mL). Similar results were obtained for (P) T16 (IC50_Viab_ = 16 µg/mL and IC50_Tox_ = 30 µg/mL) and for (Q) T188 (IC50_Viab_ =3 µg/mL and IC50_Tox_ = 10 µg/mL). Four technical replicates were used for each condition, data are representative of two independent experiments. Scale bars = 100 µm.

We have previously shown that the soluble fragment of LRIG1 (sLRIG1, Figure 1A) inhibits GBM proliferation in vivo and in vitro in different GBM cells and patient-derived orthotopic xenografts (PDOX), irrespective of their EGFR status.^22^ We established a GBM cell line expressing sLRIG1 (Supplementary Figure S1A) that presented a significant proliferation defect (Figure 1B), which was associated with a dramatic reduction in EGFRvIII protein levels (Figure 1C). To further validate the effect of sLRIG1 in other GBM cell types, we generated a purified recombinant human sLRIG1 protein (rh-sLRIG1), allowing cell treatment in a reproducible and standardized fashion (human IgG was used as a negative control protein). Folding of rh-sLRIG1 was confirmed (Supplementary Figure S1B and C), consistent with the published X-ray structure of the LRIG1 ectodomain.^27^ We applied rh-sLRIG1 to U87 cell lines with different EGFR status and verified that rh-sLRIG1 was captured by cells under treatment (Supplementary Figure S1D and E). After 6 days of treatment, we observed a significant reduction in cell number compared with control, in U87-EGFRvIII, U87-EGFR, and U87 (Figure 1D–F). In agreement with our previous results, EGFRvIII levels were significantly reduced upon treatment with rh-sLRIG1 (Figure 1G). In contrast, the level of wild-type EGFR was unchanged in U87-EGFR (Figure 1H) and slightly increased in U87 (Figure 1I). Similar to what was observed for full-length LRIG1,^28^ rh-sLRIG1 seems to affect more strongly on EGFRvIII expression compared with wild-type. EGFRvIII downregulation was concentration-dependent and most prominent 6 days after treatment, with 15 µg/mL giving the strongest effect (Supplementary Figure S1F and G). We further validated the anti-proliferative activity of rh-sLRIG1 in patient-derived GBM stem-like cells (GSCs), growing as spheres in serum-free medium (Figure 1J). After 6 days of rh-sLRIG1 treatment, proliferation was significantly reduced in NCH465 (Figure 1J and K) and NCH601 (Figure 1J and L). EGFR expression remained unchanged in NCH465 (Figure 1M), and undetectable in NCH601 (Figure 1N), further supporting an EGFR-independent effect of sLRIG1. Similar to U87-EGFRvIII cells, the strongest effect was seen with 15 µg/mL rh-sLRIG1 (Supplementary Figure S1H and I). We validated the growth-inhibitory activity of rh-sLRIG1 in different patient-derived organoids, derived from patients P3 (no EGFR amplification), T16 (EGFR-amplified) and T188 (EGFR-amplified), treated for 6 days in vitro before measuring Viability/Cytotoxicity. We observed that rh-sLRIG1 reduced the luminescence signal emitted from viable cells (referred to as “viability”) in P3, but also in T16 and T188, in a concentration-dependent manner (Figure 1O–Q). We attributed this effect to a reduced cell proliferation, since rh-sLRIG1 hardly induced cell death (referred to as “cytotoxicity”) below 30 µg/mL. Altogether, these results validate rh-sLRIG1 as an efficient compound endowed with antiproliferative activity against GBM, and suitable for soluble application. The EGFR-independent effect was in line with our in vivo study,^22^ and prompted us to ask whether other RTKs might be involved in the functional impact of sLRIG1.

### sLRIG1 Downregulates Multiple RTKs in GBM Cells, Including AXL

**Figure 2.**
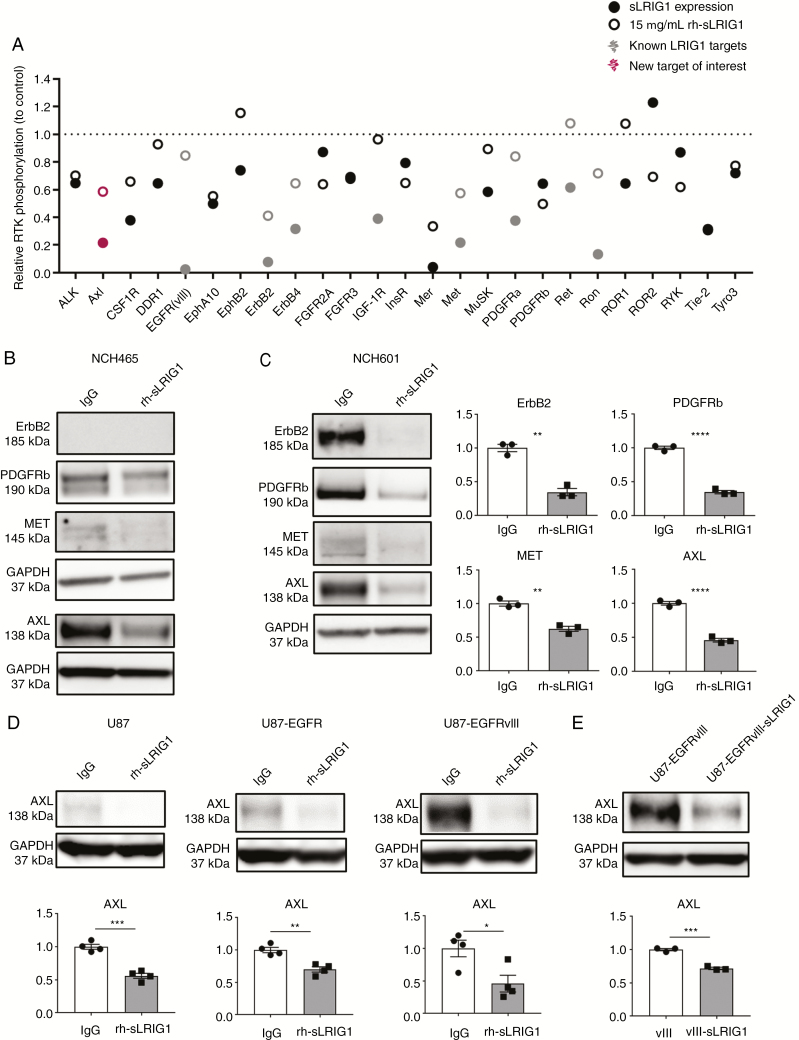
Soluble LRIG1 downregulates multiple RTKs, including AXL. (A) Phospho-RTK antibody arrays were probed with extracts of U87-EGFRvIII, U87-EGFRvIII-sLRIG1, or U87-EGFRvIII treated with 15 µg/mL rh-sLRIG1, and revealed a general decrease in RTK phosphorylation upon sLRIG1 expression/treatment. (B) Western-blot showed a significant reduction in total RTK levels after rh-sLRIG1 treatment, in NCH465 and especially in (C) NCH601: ErbB2 (*n* = 3; *P* = .001), PDGFRβ (*n* = 3; *P* < .0001), Met (*n* = 3; *P* = .0022), and AXL (*n* = 3; *P* = .0001). (D) rh-sLRIG1 reduced AXL protein levels in U87 (*n* = 4; *P* = .0002), U87-EGFR (*n* = 4; *P* = .002), U87-EGFRvIII (*n* = 4; *P* = .024). (E) AXL levels were reduced upon sLRIG1 expression (*n* = 3; *P* = .0003).

We performed a human phospho-RTK antibody array and observed that sLRIG1 expression or treatment with rh-sLRIG1 both reduced phosphorylation of known LRIG1 targets and numerous additional RTKs (Figure 2A; Supplementary Figure S1J and K). Western blot analysis showed that total protein level of ErbB2, Met, but also novel LRIG1 targets, e.g., PDGFRβ and AXL receptors were significantly reduced in NCH465 and NCH601 upon treatment with rh-sLRIG1 (Figure 2B and C). Noteworthy, AXL belongs to the TAM family of receptors (Tyro3-AXL-Mer),^29^ is involved in epithelial-to-mesenchymal transition (EMT) in cancer,^30^ and is described as a regulator of cell migration/invasion, especially in GBM.^31,32^ Protein analysis showed that AXL levels were significantly downregulated by rh-sLRIG1 addition in U87-derived cells, independent of their EGFR status (Figure 2D), and also upon sLRIG1 expression (Figure 2E). These results identify multiple novel LRIG1 targets. Among them, AXL receptor is downregulated at the protein level, independent of EGFR expression in the cells.

### sLRIG1 Alters Cytoskeleton Organization and Reduces Cell Adhesion and Invasion In Vitro

**Figure 3.**
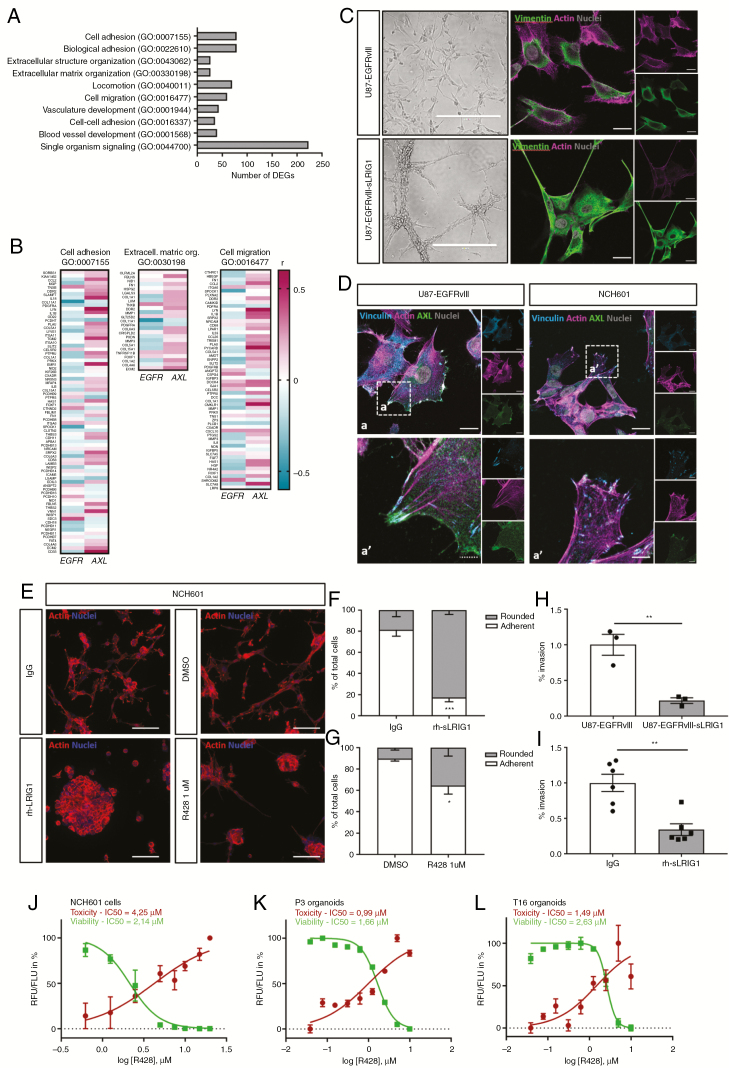
Soluble LRIG1 alters cell adhesion, cytoskeleton organisation, and reduces invasion *in vitro.* (A) A comparative genome-wide expression analysis revealed 750 DEGs upon sLRIG1 expression in U87-EGFRvIII cells, and gene ontology analysis via WebGESTALT identified the enriched biological processes. (B) Analysis of TCGA data indicated a differential correlation of *AXL* and *EGFR* expression with the genes included in these GO terms (R > 0 in red, R < 0 in blue), in GBM patients (GBM-LGG cohort, RNAseq, GBM histology subgroup, *n* = 156). (C) U87-EGFRvIII-sLRIG1 cells formed clusters and showed elongated extensions (scale bar, left panels = 400 µm), redistribution of vimentin (green), and collapse of the actin cytoskeleton (violet) (scale bar, right panels = 20 µm). (D) Immunostainings indicated colocalization of AXL (green) with vinculin (blue) at the tip of actin filaments (violet) in U87-EGFRvIII, and NCH601 cells (scale bars: a = 20 µm, a′ = 10 µm). (E) A change in adhesion upon rh-sLRIG1 or R428 treatment was shown by actin staining in NCH601 (scale bar = 100 µm). (F) Quantification of adherent/rounded cells showed a reduced adhesion after rh-sLRIG1 treatment (*n* = 3; *P* = .001). (G) A similar although lower reduction in adhesion was observed after R428 treatment (*n* = 3; *P* = .037). (H) Boyden chamber assay (16 h) demonstrated a reduced invasion of sLRIG1 expressing cells, compared with U87-EGFRvIII (*n* = 3; *P* = .0067). (I) A similar reduction in invasion was observed after 6 d of treatment with rh-sLRIG1 (*n* = 6; *P* = .0012). Viability/Cytotoxicity assay shows a concentration-dependent effect of R428 on (J) NCH601, (K) P3 organoids, and (L) T16 organoids.

To investigate the genome-wide impact of sLRIG1, we performed transcriptomic analysis of sLRIG1 overexpressing cells. From 750 differentially expressed genes (DEGs) (FDR ≤ 0.01, fold change ≥ |2|), the top DEGs were validated by qPCR (Supplementary Figure S2A–K). RTKs known to be targeted by LRIG1 were not downregulated at the mRNA level, neither after sLRIG1 overexpression (Supplementary Figure S2L) nor after rh-sLRIG1 treatment (Supplementary Figure S2M–O). Gene ontology (GO) analysis of the protein-coding DEGs by WebGeSTALT indicated (1) cell adhesion, (2) extracellular matrix organization, and (3) migration as the main biological processes influenced by sLRIG1 (Figure 3A; Supplementary Figure S3A). Heatmaps displaying DEGs in these enriched biological processes showed clear distinct clustering (Supplementary Figure S3B–D). The analysis of TCGA data via the GlioVis platform^33^ showed that in GBM patients, *AXL* expression is highly correlated with most of the genes that are included in these GO categories, whereas *EGFR* shows a contrasting correlation profile (Figure 3B). These results corroborated the morphological changes observed in sLRIG1-expressing cells: they displayed a more condensed shape and formed clusters. We observed a reorganization of vimentin filaments, and a collapse of the actin cytoskeleton (Figure 3C). Based on the involvement of AXL in cell adhesion and invasion, we analyzed AXL contribution to GBM cell morphology and confirmed its colocalization with vinculin at the tip of actin filaments (Figure 3D). AXL staining was associated with actin at cell protrusions in GBM cells, which were lost in the presence of sLRIG1 (Supplementary Figure S4A and B). We also studied the effect of rh-sLRIG1 on the EGFR-negative GSC line NCH601 plated on ECM by quantifying the number of low-adherent (rounded) versus adherent cells. We found that rh-sLRIG1 treatment induced a dramatic change in cell adhesion compared with IgG (Figure 3E and F), recapitulating the previous results. A similar albeit lower effect was observed upon treatment with the AXL inhibitor R428 compared with DMSO (Figure 3E and G). These data prompted us to ask whether sLRIG1 could also affect GBM cell invasion. We found that sLRIG1 overexpression dramatically impaired cell invasion through an ECM-coated membrane (Figure 3H), and this effect was reproduced after treatment with rh-sLRIG1 (Figure 3I). Together with recent reports,^34,35^ these data endorse AXL as a regulator of cell motility, proliferation, and survival, and suggest that AXL might be involved in the cell remodelling observed upon sLRIG1 treatment. We further confirmed that AXL inhibition by R428 potently reduced cell viability in a concentration-dependent manner, in NCH601 cells and in P3 and T16 patient-derived organoids (Figure 3J–L).

### At the Molecular Level, sLRIG1 Interferes With AXL But Not With EGFR Dimerization

**Figure 4.**
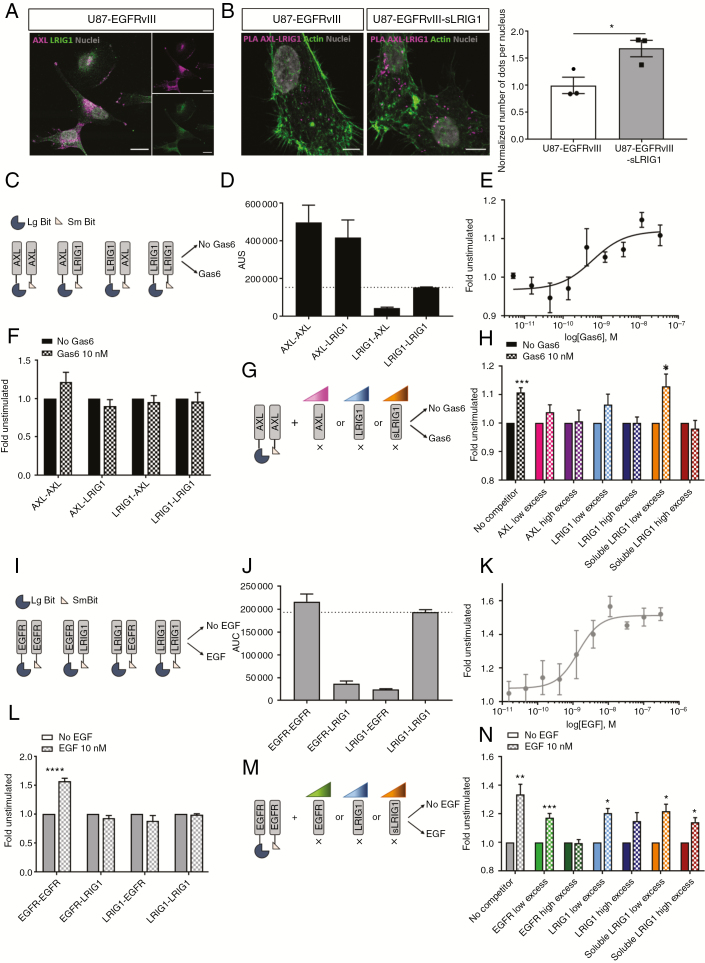
Soluble and full-length LRIG1 hinder ligand-induced dimerization of AXL but not of EGFR. (A) Immunofluorescent staining of AXL (violet) and LRIG1 (green) in U87-EGFRvIII, indicating colocalization (scale bar = 20 µm). (B) In situ proximity ligation assay for AXL and LRIG1 (scale bar = 20 µm). The relative number of interaction foci was increased upon sLRIG1 expression (*n* = 3; *P* = .033). (C) Design of NanoBiT complementation assay for the study of LRIG1-AXL interaction. (D) The area under the curve (AUC) provided by the complementation was high for AXL–AXL coupling, but also for AXL–LRIG1, suggesting interaction. (E) Gas6 increased AXL dimerization in a concentration-dependent way (*n* = 3; EC50 = 0.6 nM). (F) Application of 10 nM Gas6 (15 min) recapitulated AXL dimerization (*n* = 3; *P* = .010) but did not modify the signal corresponding to AXL–LRIG1, LRIG1–AXL, or LRIG1–LRIG1. (G) Competition experiments were designed to monitor AXL dimerization in the presence of nontagged competitors. (H) AXL dimerization was maintained in absence of competitor (*n* = 4; *P* = .0008), but reduced in presence of 2X (*n* = 4; *P* = .19) and 9X (*n* = 4; *P* = .89) unlabeled AXL. Dimerization was abolished in presence of 2X (*n* = 4; *P* = .13) and 9X (*n* = 4; *P* = 0.96) LRIG1. Upon 2X soluble LRIG1, AXL dimerization was still significant (*n* = 4; *P* = .026), but back to baseline upon 9X soluble LRIG1 (*n* = 4; *P* = .522). (I) Design of NanoBiT complementation study of LRIG1 interaction with EGFR. (J) The AUC was high for EGFR–EGFR coupling, but not for EGFR–LRIG1 nor LRIG1–EGFR. (K) EGF increases EGFR dimerization in a concentration-dependent way (*n* = 3; EC50 = 1.40 nM). (L) Application of 10 nM EGF (10 min) recapitulated EGFR dimerization (*n* = 3; *P* < 0.0001) but did not modify the signal for EGFR–LRIG1, LRIG1–EGFR, or LRIG1–LRIG1. (M) Competition experiments were applied to monitor EGFR dimerization in the presence of non-tagged competitors. (N) Dimerization was maintained in absence of competitor (*n* = 7; *P* = .003) and in presence of 4X EGFR, (*n* = 7; *P* = .0006) but abolished upon 18X (*n* = 7; *P* = .82) EGFR competitor. EGFR dimerization was maintained in presence of 4X (*n* = 4; *P* = .033) and 9X (*n* = 4; *P* = .08) LRIG1. Similarly, dimerization was significant upon 4X (*n* = 4; *P* = .016) and 18X (*n* = 4; *P* = .016) soluble LRIG1.

Immunofluorescence staining indicated colocalization of AXL with LRIG1 in GBM cells (Figure 4A). By in situ proximity ligation assay, we observed a relative increase in the number of interaction foci in sLRIG1 expressing cells, confirming a close interaction between both proteins (Figure 4B). Based on the observation that AXL was downregulated by LRIG1, whereas EGFR was not, we aimed to determine the differential mechanism of action. We assessed the real-time interaction between LRIG1 and RTKs, using a nanoluciferase-based complementation assay (NanoBiT, Promega)^24^ in GBM cells transfected with plasmids encoding for AXL or LRIG1, both C-terminally fused to a part of the luciferase enzyme (LgBiT or SmBiT; Figure 4C). As reported previously,^36^ AXL–AXL interaction (dimerization) generated a strong signal, even in absence of ligand. Of note, the AXL-LRIG1 coupling also generated a strong signal, indicating putative protein–protein interaction. Such signal was not observed in the LRIG1–AXL configuration, which might be explained by the different sizes of the C-terminal tails of the two proteins fused to the fragments of the split nanoluciferase (Figure 4D; Supplementary Figure S5A). In the presence of Gas6, AXL dimerization increased in a concentration-dependent manner (Figure 4E and F), but no ligand-induced increase in AXL-LRIG1 interaction was recorded for the two configurations tested (Figure 4F). To circumvent the possible bias linked to the size of the C-terminal tails of the proteins for efficient complementation, we monitored AXL dimerization in the presence of nontagged competitors that were introduced in increasing concentration (Figure 4G). In the presence of untagged AXL monomer in excess, the Gas6-induced signal increase could no longer be observed, indicating an interference with dimerization of the tagged receptors. Similar results were obtained with an excess of LRIG1, as well as in the presence of sLRIG1 (the secreted form of LRIG1; Figure 4H; Supplementary Figure S5B). These results indicate that both sLRIG1 and full-length LRIG1 directly interact with AXL in a ligand-independent manner, resulting in impaired AXL dimerization. We applied the same approach to determine LRIG1 interaction with EGFR (Figure 4I). Without EGF, we observed ligand-independent dimerization of EGFR.^37^ However, the low signal for EGFR-LRIG1 interaction indicated a very low rate of molecular encountering (Figure 4J), despite both proteins being expressed after transfection (Supplementary Figure S5C). In the presence of EGF, we observed a concentration-dependent increase in EGFR dimerization (Figure 4K and L), which was not observed for EGFR-LRIG1 and other couplings (Figure 4L). The signal corresponding to EGFR dimerization was abrogated upon excess of untagged EGFR competitor, but not affected by increasing amounts of LRIG1 or sLRIG1 (Figure 4M and N), indicating that they do not interfere with EGFR dimerization. In summary, we demonstrate a direct protein–protein interaction of LRIG1 (and sLRIG1) with AXL, but not with EGFR, suggesting that LRIG1-induced downregulation of RTKs requires molecular interactions.

## Discussion

As a negative regulator of RTKs, in particular EGFR and other members of the ErbB family, LRIG1 represents a promising anticancer agent for various malignancies, including GBM. Our previous work showed that interstitial delivery of the soluble part of LRIG1 (sLRIG1) potently inhibited GBM growth in vivo independent of EGFR status,^22^ suggesting that additional RTKs may be involved. In this study, we introduce recombinant human sLRIG1 protein (rh-sLRIG1) as an active protein, suitable for soluble application. We show that rh-sLRIG1 efficiently affects growth and adhesion of GBM cells and patient-derived organoids, and has *pan*-RTK inhibition activity. We highlight AXL as a novel sLRIG1 target and show a direct LRIG1-AXL interaction that appears to be required for receptor regulation.

Our findings enlarge the panel of LRIG1 targets to 10 RTK members including EGFR, ErbB2, ErbB3, ErbB4, Met, Ret, Ron, PDGFRα, IGF1R^14,38^ and AXL, non-TK receptors,^39^ and tumor-specific RTKs such as EGFRvIII. A previous report showed that LRIG1 expression induced a stronger downregulation of EGFRvIII protein levels compared with EGFR wild-type,^28^ which we confirm here with rh-sLRIG1 treatment. The sensitivity of EGFRvIII to sLRIG1 contrasts with its resistance to small molecule inhibition,^40^ and likely attributable to its truncated ectodomain, possibly responsible for a better interaction with sLRIG1, or to specific signaling related to its constitutive activity.^41^ Along the same line, a recent study showed that the tumor-suppressive effect of LRIG1 was stronger in lung cancer cells harboring EGFR mutations, compared with EGFR wild-type cells.^38^ Our results show that sLRIG1 does not interact with EGFR, but its interaction with EGFRvIII could not be firmly tested in our assay (this receptor does not respond to ligand). In the case of AXL, our results strongly correlate the protein–protein interaction (and interference with receptor dimerization) with the RTK downregulation at the protein level. We speculate that (soluble) LRIG1 exerts a receptor-specific anticancer activity that might be depending on receptor sequence, 3D-structure, or third-party interactors. The signaling events that occur downstream of LRIG1-RTK interactions can therefore be diversified accordingly and need to be comprehensively untangled.

We show that rh-sLRIG1 not only affects cell proliferation but impairs adhesion and invasion. The cytoskeleton plays a pivotal role in cell adhesion, intracellular organization, differentiation, and division, and cytoskeleton defects directly affect cell survival.^42^ RTKs are recognized modulators of actin dynamics and other cytoskeleton proteins. Among them, AXL modulates actin polymerisation, promotes cell-matrix adhesion, connects with focal adhesions in lung cancer, Schwannoma or GBM cells,^34,35,43^ and is generally associated with a migratory phenotype. Here, we confirm that AXL inhibition affects GBM cell adhesion, which may underlie the sLRIG1 effect, although at this stage we cannot rule out additional AXL-independent effects. Our data also show that sLRIG1 affects GBM cell invasion, but the extent of RTK involvement remains to be elucidated. EGFR, EGFRvIII, and AXL contribute to GBM invasion (either individually or in combination),^4,44^ while concurrently affecting other cell phenotypes.^45^

The redundancy of RTK signaling and the development of resistance to targeted RTK inhibitors are well-known challenges in cancer. Several reports indicated AXL as a major driver of resistance to anti-EGFR therapies, e.g., in lung cancer^46^ and in GBM.^47^ At the molecular level, AXL-EGFR heterodimerization or transactivation diversify downstream signaling into additional pathways, beyond those triggered by individual receptors, which limits the efficacy of EGFR targeting strategies^44,48^ and stresses the potential of AXL inhibition in the treatment of EGFR-driven GBM. With regard to the dismal outcome of RTK-targeting in GBM, a broader perspective on RTK inhibition could be considered. *Pan*-RTK targeting based on drug combination approaches,^49^*Pan*-ErbB inhibitors,^50^ or antibodies^51^ was shown to overcome resistance and to suppress tumorigenesis more efficiently than single receptor targeting. We have demonstrated that sLRIG1 is endowed with *pan*-RTK inhibitory activity, and may be a valid candidate in this context. Ultimately, a better insight in the molecular determinants of sLRIG1 activity will help us to design a more effective and clinically suitable sLRIG1-based therapeutic against RTK-dependent cancers.

## Funding

This work was supported by Télévie Belgium-Luxembourg (7464715, 7463117, 7456814, 7461515 [to V.N. and S.P.N.]), Luxembourg National Research Fund (Nanokine-15/10358798, AFR-3004509, and PRIDE-11012546 “NextImmune” [to M.S., M.M., and A.C.]), and German Cancer Consortium (DKTK), German Research Foundation (DFG) Collaborative Research Centers 1080 (A03) and 1292 (TP09) to M.H.H.S. This work was funded by the Télévie Belgium-Luxembourg (7.4647.15, 7.4631.17, 7.4568.14, and 7.4615.15, 7.8513.18), the Luxembourg National Research Fund (Nanokine-15/10358798, AFR-3004509, and PRIDE-11012546 “NextImmune”), and the German Cancer Consortium and the German Research Foundation Collaborative Research Centers 1080 (A03) and 1292 (TP09).

## Supplementary Material

vdz024_suppl_Supplementary_Figure_Legends

vdz024_suppl_Supplementary_Figure_S1

vdz024_suppl_Supplementary_Figure_S2

vdz024_suppl_Supplementary_Figure_S3

vdz024_suppl_Supplementary_Figure_S4

vdz024_suppl_Supplementary_Figure_S5

vdz024_suppl_Supplementary_Methods

vdz024_suppl_Supplementary_Table_S1

## Notes

**Authorship statement**: Experimental design: V.N., M.H.H.S., and S.N. Experimental work: V.N., A.H., A.S., K.T., E.K., A.M., M.S., and M.M. Data analysis: V.N., A.H., S.F., P.V.N., A.M., and A.C. Data interpretation: V.N., A.H., A.S., S.F., A.C., and S.N. Manuscript writing: V.N., A.H., A.S., S.F., K.T., P.V.N., A.M., M.S., M.M., A.C., M.H.H.S., and S.N.
