# Supplementary material for: The soluble form of pan-RTK inhibitor and tumor suppressor LRIG1 mediates downregulation of AXL through direct protein–protein interaction in glioblastoma
Source: Neurooncol Adv. 2019 Sep 6;1(1):vdz024. doi: 10.1093/noajnl/vdz024 (PMC7212925; doi:10.1093/noajnl/vdz024)
Supplement: vdz024_suppl_Supplementary_Figure_Legends [file vdz024_suppl_supplementary_figure_legends.docx]

**Supplementary figure legends**

**Supplementary figure S1 – Characterization of recombinant human sLRIG1 (rh-sLRIG1).**

(A) Quantitative real-time PCR showed that U87-EGFRvIII-sLRIG1 have increased LRIG1 mRNA expression, compared to U87-EGFRvIII (n=3, p=0.0396). (B) Far-UV CD spectrum of rh-sLRIG1. (C) Intrinsic fluorescence emission. A significant blueshift of the wavelength corresponding to maximum tryptophan emission (λ_max_ = 330 nm) indicated subtantial tertiary contacts. (D) For localization of rh-sLRIG1 or IgG, we used His-tag antibody. Immunostainings (green) showed that GBM cells have specifically captured rh-sLRIG1 after 6 days of treatment, whereas IgG was almost not retained (scale bar = 20 µm). (E) Co-staining of His-tag with endosomal markers Rab5A and EEA1 showed partial colocalization of rh-sLRIG1 with endosomes (scale bars = 10 µm /5 µm). (F) Western-blot analysis showed a concentration-dependent reduction of EGFRvIII and AXL protein levels upon rh-sLRIG1 treatment, especially at 15 µg/mL. (G) Time-course analysis showed that EGFRvIII protein levels were altered after 6 days of treatment. (H) Growth curves were calculated for NCH465, and indicate that only 15 µg/mL rh-sLRIG1 impact cell growth *in vitro*, when compared to IgG (n=3. At day 7: p=0.0183; at day 10: p<0,0001). (I) This was associated with a decrease of AXL protein levels, as shown by Western-blot. (J) Phospho-RTK antibody arrays were probed with extracts of U87-EGFRvIII, U87-EGFRvIII-sLRIG1 or U87-EGFRvIII treated with 15 µg/mL rh-sLRIG1 for 6 days. (K) Total levels of PDGFRb and Met were decreased in U87-EGFRvIII treated with 15 µg/mL rh-sLRIG1 for 6 days.

**Supplementary figure S2 – Validation of the top up/downregulated genes isolated from the microarray analysis.**

(A) A heatmap displays the top up- and downregulated genes isolated from the microarray analysis comparing U87-EGFRvIII and U87-EGFRvIII-sLRIG1. (B-K) Several genes were validated by quantitative real-time PCR analysis (n=3 in each group. ** = p<0,01; *** = p<0,001; **** = p<0,0001). (L) Genes encoding for RTKs known to be targeted by LRIG1 were not regulated upon sLRIG1 expression. (M-O) qPCR confirmed that EGFR, AXL or Met were not regulated upon 15 µg/mL rh-sLRIG1 treatment.

**Supplementary figure S3 – sLRIG1 is associated with differentially expressed genes belonging to specific biological processes.**

(A) Based on microarray analysis, we identified 750 genes that were differentially expressed upon sLRIG1 expression in U87-EGFRvIII, and used WEB-based Gene SeT AnaLysis Toolkit (WebGeSTALT) for data analysis. Heatmaps display the top regulated genes corresponding to enriched biological processes, respectively (B) adhesion (GO:0007155), (C) cell migration (GO:0016477) and (D) extracellular matrix organization (GO:0030198), which all show a clear clustering of the experimental groups.

**Supplementary figure S4 – rh-sLRIG1 reduces AXL staining at cell protrusions.**

(A) Immunofluorescent stainings showed that AXL (green) colocalized with actin (violet) at cell protrusions (indicated by arrows), but these were lost upon sLRIG1 expression. (B) Treatment with rh-sLRIG1 recapitulated this loss of AXL/actin staining (green/violet), and cell protrusions were unclear. Scale bars = 20 µm.

**Supplementary figure S5 – Additional results on LRIG1-RTK interactions.**

(A) Representation of the area under the curve calculation, based on the luminescence signal recorded for 20 minutes. (B) Immunofluorescence stainings demonstrated that AXL (violet) and LRIG1 (green) were colocalized at the cell membrane in U87 cells upon transfection. Conversely, soluble LRIG1 was not found at the membrane, but rather in the Golgi apparatus (and most likely secreted). (C) Similar results were obtained for EGFR and LRIG1. Scale bars = 20 µm.
