# Supplementary figures and images for: The soluble form of pan-RTK inhibitor and tumor suppressor LRIG1 mediates downregulation of AXL through direct protein–protein interaction in glioblastoma
Source: Neurooncol Adv. 2019 Sep 6;1(1):vdz024. doi: 10.1093/noajnl/vdz024 (PMC7212925; doi:10.1093/noajnl/vdz024)

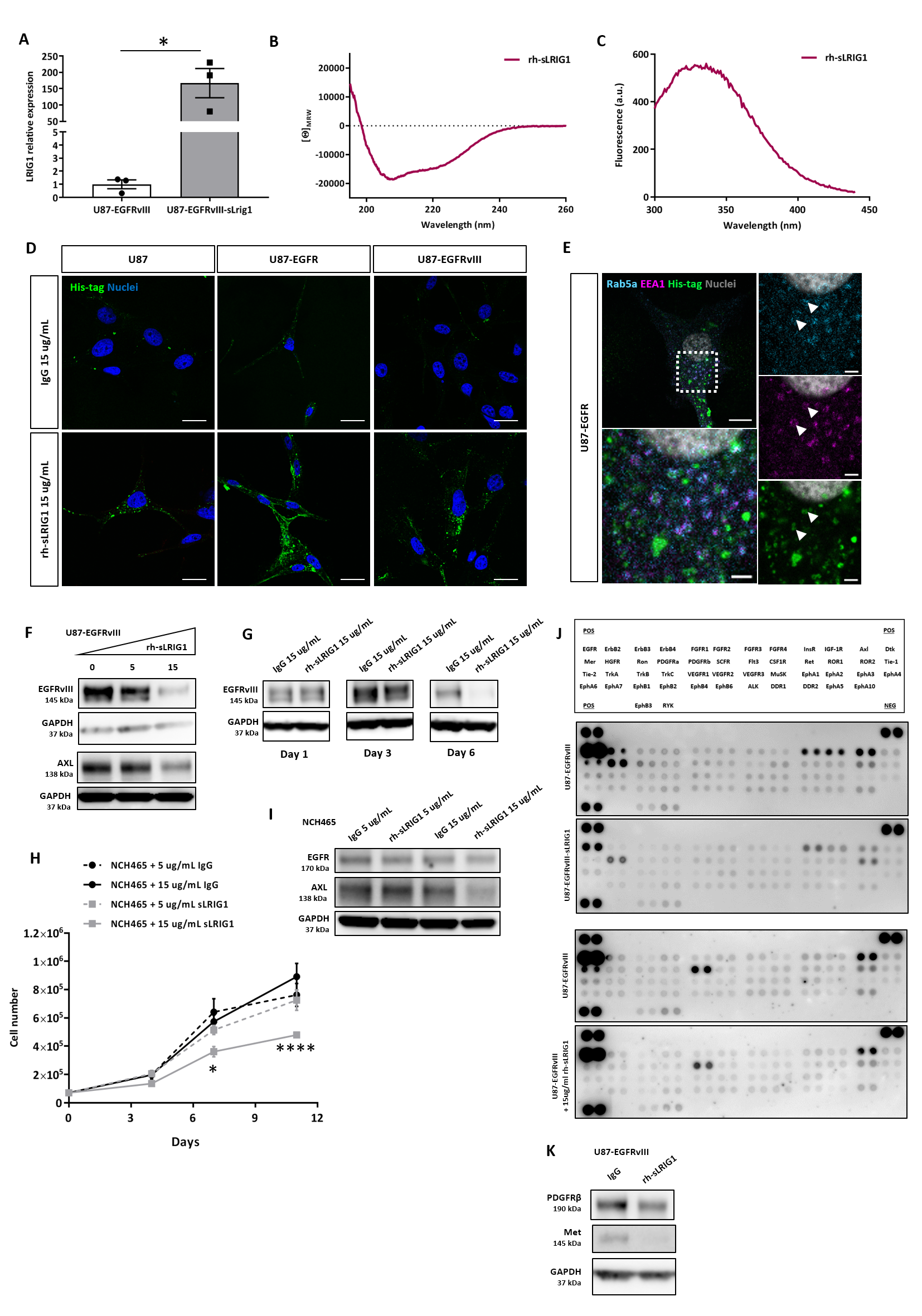

Supplement: vdz024_suppl_Supplementary_Figure_S1 [file vdz024_suppl_supplementary_figure_s1.png]

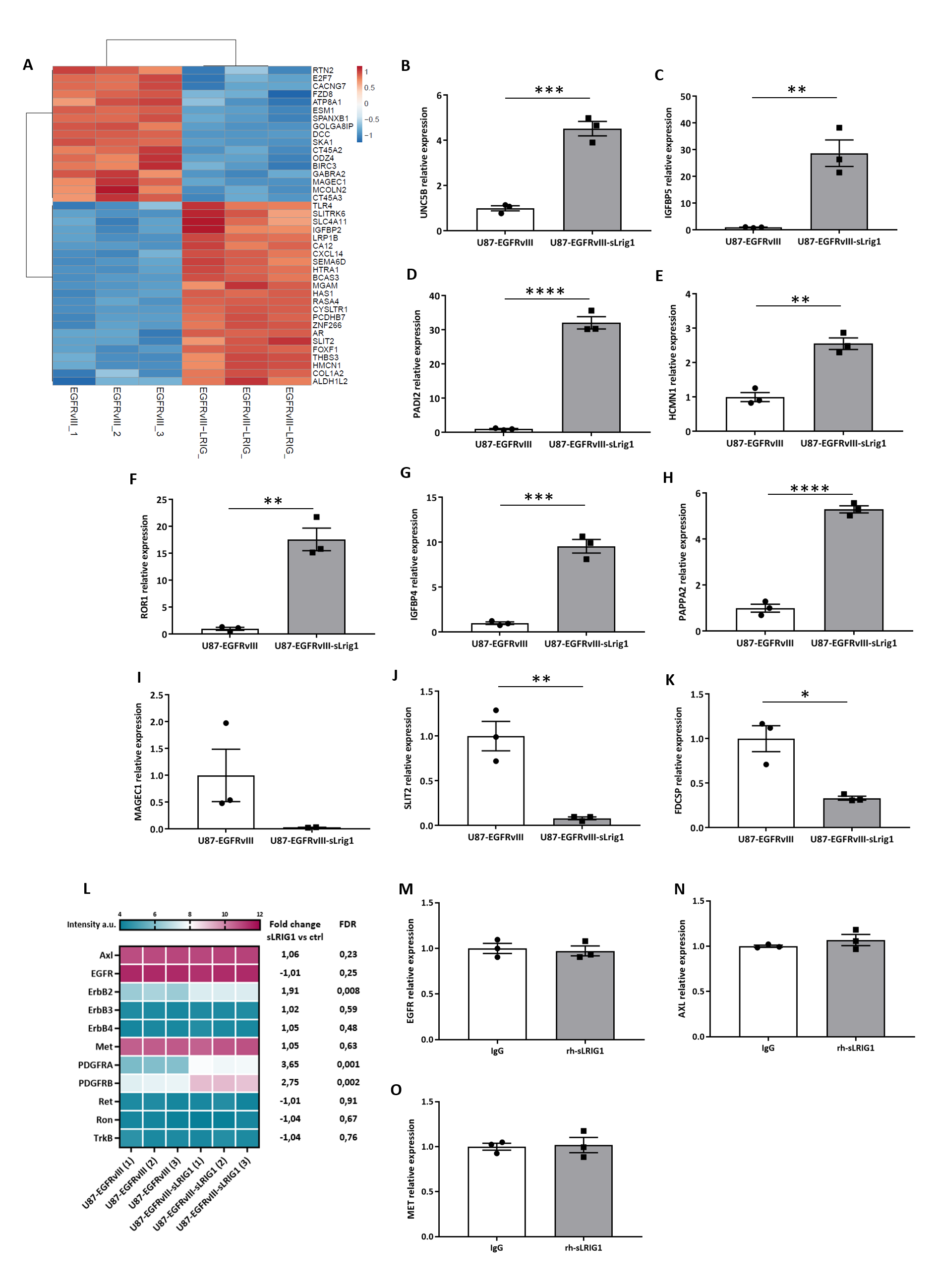

Supplement: vdz024_suppl_Supplementary_Figure_S2 [file vdz024_suppl_supplementary_figure_s2.png]

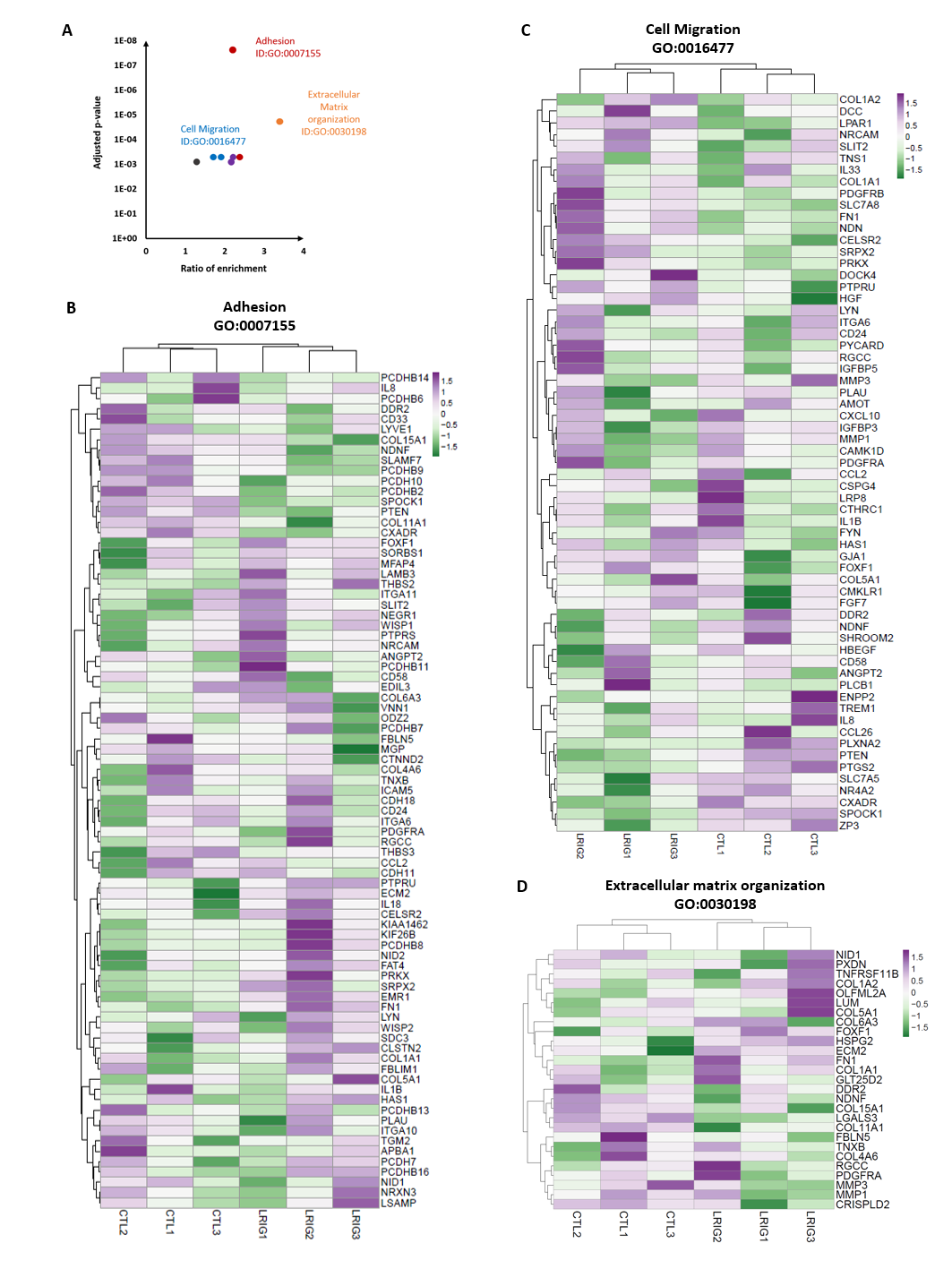

Supplement: vdz024_suppl_Supplementary_Figure_S3 [file vdz024_suppl_supplementary_figure_s3.png]

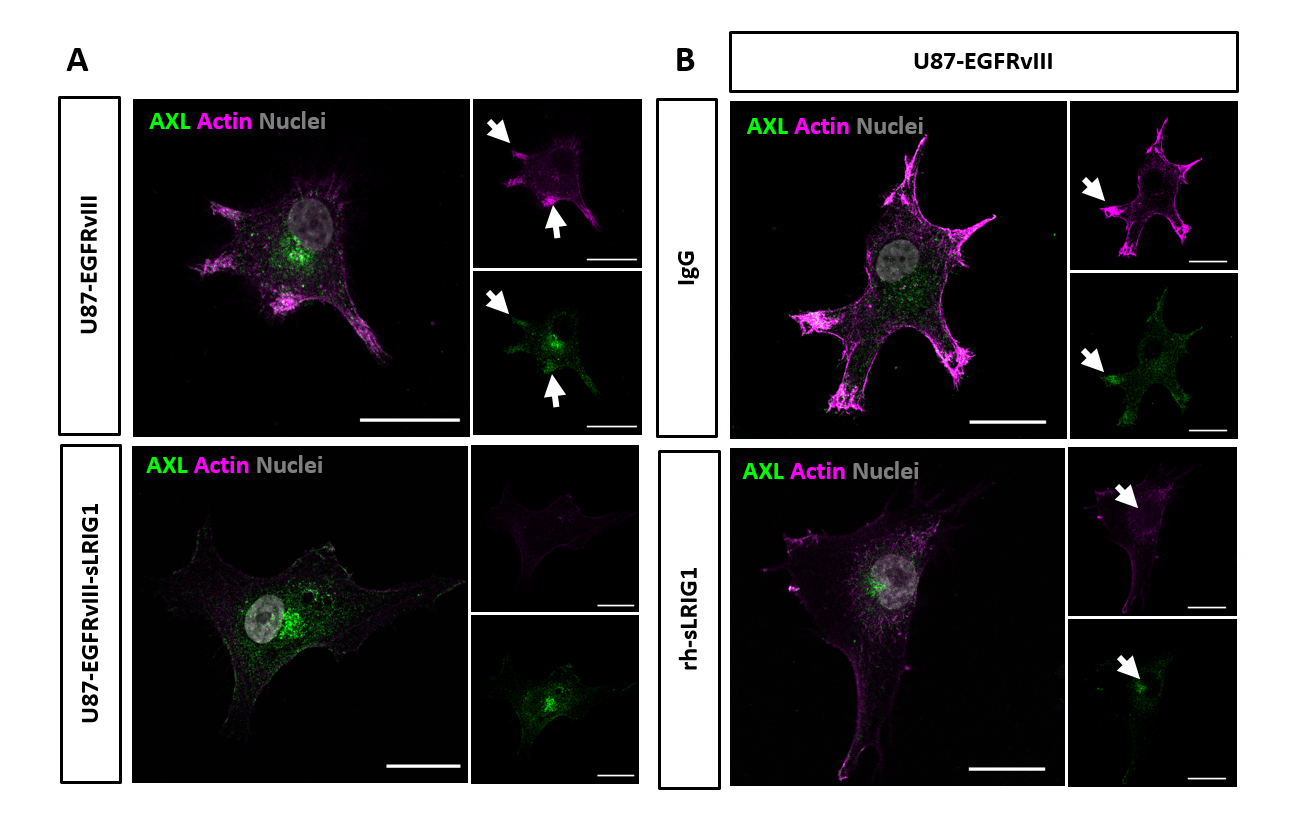

Supplement: vdz024_suppl_Supplementary_Figure_S4 [file vdz024_suppl_supplementary_figure_s4.png]

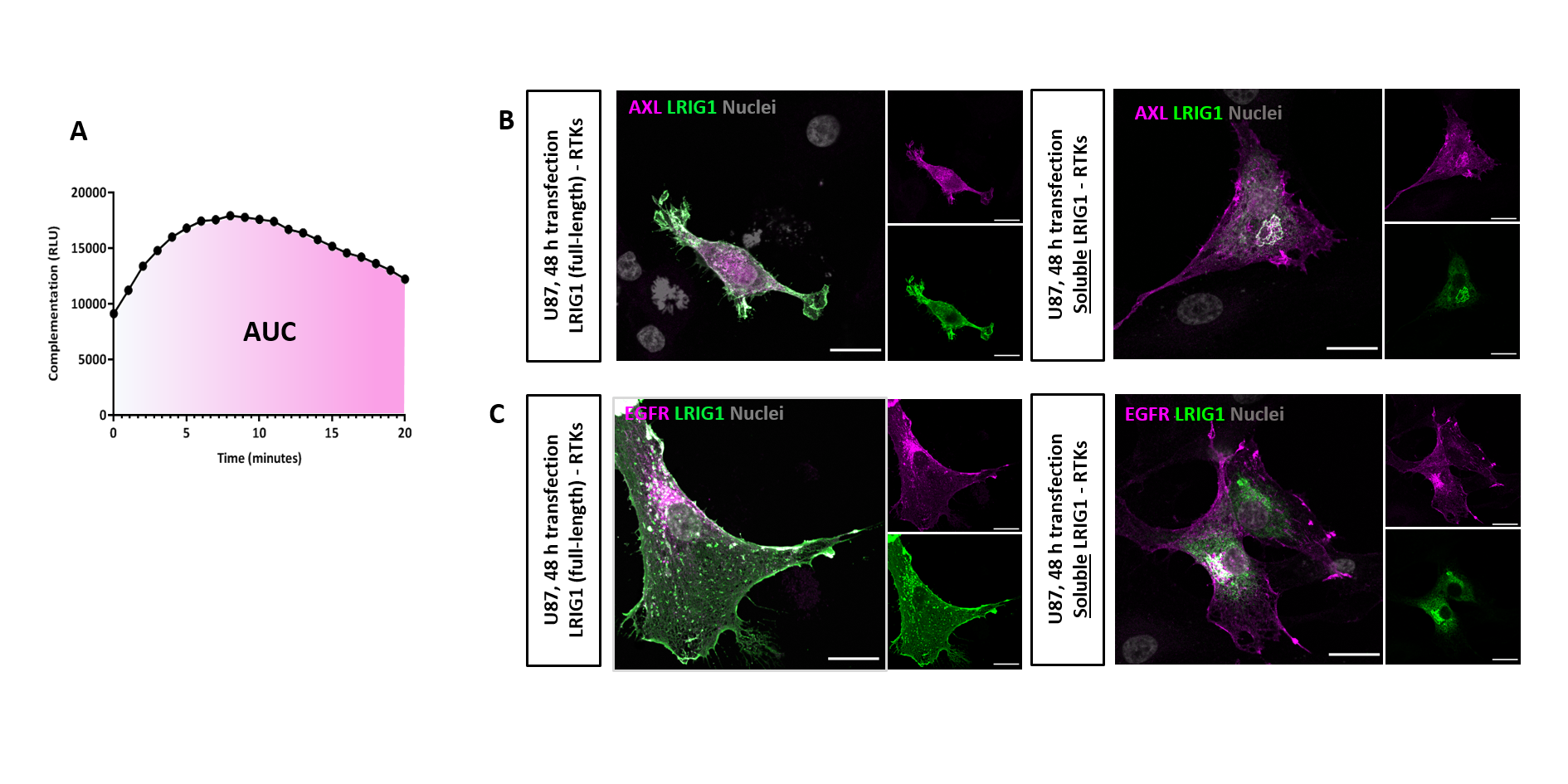

Supplement: vdz024_suppl_Supplementary_Figure_S5 [file vdz024_suppl_supplementary_figure_s5.png]
