# Supplementary material for: The soluble form of pan-RTK inhibitor and tumor suppressor LRIG1 mediates downregulation of AXL through direct protein–protein interaction in glioblastoma
Source: Neurooncol Adv. 2019 Sep 6;1(1):vdz024. doi: 10.1093/noajnl/vdz024 (PMC7212925; doi:10.1093/noajnl/vdz024)
Supplement: vdz024_suppl_Supplementary_Methods [file vdz024_suppl_supplementary_methods.docx]

**Supplementary methods**

**Circular dichroism (CD) and fluorescence spectroscopy.** Far-UV CD spectra (195-260 nm) were recorded with a Jasco J-810 spectropolarimeter at 20°C in Milli-Q water, using a 1 mm pathlength quartz Suprasil cell (Hellma), with a protein concentration of 0.1 mg/ml (1.15 µM). Four scans (10 nm/min, 1 nm bandwidth, 0.2 nm data pitch and 1s DIT) were averaged, base lines were subtracted and no smoothing was applied. Data are presented as the molar residue ellipticity ([Ɵ]_MRW_). Intrinsic fluorescence spectrum was recorded with a Cary Eclipse spectrofluorimeter (Varian), using a 1 cm pathlength cell and bandwidths of 5 nm for both excitation and emission, with a protein concentration of 0.01 mg/ml (0.115 µM). The excitation wavelength was 280 nm and emission was recorded in the 300-440 nm range, at a rate of 600 nm·min-1. Five spectra were accumulated and averaged.

**Viability and cytotoxicity assays**. PDOX-derived cells (P3, T16 and T188) were freshly isolated and seeded in 384-well plates. After spheroid formation, we applied rh-sLRIG1 or R428 (AXL inhibitor, S2841, SelleckChem) and performed CellTiter-Glo^®^2.0 and CellTox^TM^-Green assays (Promega), according to the manufacturer’s instructions. IgG and DMSO were used as negative controls. After 3 days (AXL inhibitor) or 6 days (rh-sLRIG1), CellTox reagent was added for 15 min before recording of fluorescence emission. This was followed by a 30-minutes incubation with CellTiter-Glo reagent, and luminescence was measured (ClarioStar, BMG Labtech). Analysis and IC50 determination were performed via GraphPad Prism 7 software. For LIVE/DEAD labelling, organoids were incubated with 2 mM Calcein AM and 4 mM Ethidium homodimer-1 (LIVE/DEAD assay kit, Molecular Probes) for 6 hours. Imaging was done using a LSM510 Confocal Laser microscope (Zeiss).

**Invasion assay.** Boyden chamber assays were performed using inserts (8 μm pore size, Greiner) coated with a 1:1 mixture of 0.05 mg/ml collagen type I (Sigma) and 0.5 mg/ml ECM proteins (Sigma). Cells were plated at a density of 5x10^4^ cells/insert, and incubated for 16 hours. Invaded cells on the bottom of the filter were fixed with 4% paraformaldehyde and stained with Crystal Violet. Cells were counted in five representative fields/insert, countings were corrected for proliferation to obtain the percentage of invasion.

**Gene expression analysis.** Total RNA of triplicates of each U87-EGFRvIII and U87-EGFRvIII-sLRIG1 cells was extracted using the RNeasy Mini Kit (Qiagen) and measured using the NanoDrop® ND-1000 Spectrophotometer (Software V3.7.1): Quality was examined using the Agilent RNA 6000 Nano Kit and Bioanalyzer (Agilent Technologies). Total RNA was hybridized on Human Gene 2.0 ST Arrays (Affymetrix) in accordance with the manufacturer’s instructions. Statistical analysis of signal intensities was performed using Partek Genomics Suite (Partek GS) software (version 6.13.0621 ©; 2013 Partek Inc.). A list of differentially expressed genes (DEG) was created by analysis of variance (ANOVA) with Benjamini-Hochberg`s FDR<0.01 and an absolute fold change FC>2. The WEB-based Gene SeT AnaLysis Toolkit (WebGeSTALT) was used for data mining. Raw data are accessible on GEO (E-MTAB-7474).

**Reverse transcription and qRT-PCR.** cDNA was synthesized using iScript Reverse Transcriptase (BioRad) and applied for real-time PCR reaction in a Via7 instrument using Fast SYBR Green (Applied Biosystems) and specific primers (Eurogentec) (Table S1). Relative gene expression levels were normalized against elongation factor 1 alpha (EF1α) housekeeping gene, and calculated using the ΔC_T_ method. Reactions were performed in triplicates.

**Western blot and antibody arrays.** Protein extracts were resolved in NuPage 4-12 % BisTris gels (ThermoFisher), and blotted onto a PVDF membrane according to standard protocols. Blots were probed with antibodies targeting EGFR (AHR5062, ThermoFisher), AXL (8661S, Cell Signaling Technology, CST), Actin (MAB1501, Millipore), or GAPDH (5174, CST), at 4°C overnight. Horseradish peroxidase (HRP)-coupled antibodies (Jackson ImmunoResearch) were applied for 1 hour at RT, and blots were developed with a chemiluminescent substrate (ThermoFisher). Human phospho-RTK antibody array (ARY001B, R&D Systems) was performed following the manufacturer’s instructions. Blots and arrays were imaged with the ImageQuant 350 scanning system (cooled-CCD camera, GE Healthcare), and intensity levels were quantified using ImageJ software. For western-blot results, the expression of proteins of interest is represented as the relative expression to housekeeping protein, normalized to the control condition.

**Immunofluorescence.** GBM cells were fixed with 4% paraformaldehyde and blocked for 1 hour with PBS supplemented with 0.1 % Triton X-100 (PBS-T) and 10% FBS. Antibodies specific for (s)LRIG1 (MAB7498, R&D Systems), AXL (8661, CST), His-tag (14930, CST), Rab5A (sc166622, Santa Cruz), EEA1 (sc6415, Santa Cruz), vimentin (MAB3400, Millipore), vinculin (**V9131, Sigma**), were diluted in PBS-T and incubated for 2 hours at room temperature. After PBS washing, cells were incubated with Alexa Fluor 488, 555 or 647-conjugated antibodies (ThermoFisher). Nuclei were counterstained with Hoechst, and actin filaments were stained with ActiStain®-555 (ThermoFisher). Image acquisition and analysis were performed using a LSM880 Confocal microscope and ZEN2 software (Zeiss).

***In situ* proximity ligation assay.** Proximity ligation assay (PLA) was performed to detect interaction between sLRIG1 and AXL. Cells were incubated overnight with primary antibodies specific for LRIG1 (MAB7498, R&D Systems) and AXL (8661S, Cell Signaling Technology). DuoLink® detection kit plus and minus probes for mouse and rabbit (Sigma) were used according to the manufacturer’s instructions. Nuclei were counterstained, and coverslips were mounted with FluoroMount aqueous medium (Sigma). Image acquisition was performed using Ni-E microscope (Nikon) and LSM880 Confocal microscope and ZEN2 software (Zeiss), and analysis was done with Image J software.

**NanoBiT® complementation assay**. RTK dimerization and interaction with LRIG1 were monitored by NanoLuc complementation assay (NanoBiT, Promega) ^22-24^. 1.2x10^6^ U87 cells were plated in 10-cm culture dishes and transfected with pNBe vectors containing human EGFR, AXL or LRIG1, C-terminally fused to LgBiT or SmBiT. For competition experiments, pIRES plasmids containing untagged EGFR, AXL, LRIG1 or sLRIG1 were co-transfected. 48 hours post-transfection, cells were harvested and distributed into white 96-well plates (5x10^4^ cells per well), incubated at 37°C with ligands of interest (10 min for EGF, 15 min for Gas6) and then with 200-fold diluted Nano-Glo Live Cell substrate. RTK dimerization or interaction with LRIG1 in response to ligands were evaluated with a ClarioStar luminometer (BMG LabTech). The signal is reported as a ratio to “untreated” control condition (without ligand), being set to 1.
