# Supplementary material for: The soluble form of pan-RTK inhibitor and tumor suppressor LRIG1 mediates downregulation of AXL through direct protein–protein interaction in glioblastoma
Source: Neurooncol Adv. 2019 Sep 6;1(1):vdz024. doi: 10.1093/noajnl/vdz024 (PMC7212925; doi:10.1093/noajnl/vdz024)
Supplement: vdz024_suppl_Supplementary_Table_S1 [file vdz024_suppl_supplementary_table_s1.docx]

| ***Gene*** | ***Forward primer (5’→ 3’)*** | ***Reverse primer (3’→ 5’)*** |
| --- | --- | --- |
| EF1α | TTGTCGTCATTGGACACGTAG | TGCCACCGCATTTATAGATCAG |
| FDCSP | GTTCTCCTCCTGATCACAGCC | TCAATTTCAGGTGACCAGGTTT |
| HMCN1 | TGGCCCAGTGACAATTACCAC | TGGACCGAGCATCAGTGAAAA |
| IGFBP4 | AGCCCTCTGACAAGGACGAG | GGTGCTCCGGTCTCGAATTT |
| IGFBP5 | TGACCGCAAAGGATTCTACAAG | CGTCAACGTACTCCATGCCT |
| LRIG1 | GGACTTGCCGAACCTACAGG | GCTGCGAATCTTGTTTGTGCTG |
| MAGEC1 | GCGGAGGGAGGAGACTTATAGA | GGAGAAGACTCGGCATCCCA |
| PADI2 | ACCTCTGGACCGATGTCTACA | TCCCTTCCTCGTCATAGTAGTTG |
| PAPPA2 | ACTCACCCAAGAGGGCATACA TGA | GCACTGAGCTGGCAAAGTAGATGT |
| ROR1 | CAACAAGAAGCCTCCCTAATGG | CCTGAGTGACGGCACCTAGAA |
| SLIT2 | CCAGCTTCTGCCATTTACAGTGT | TTCCCTGGCATGCCTCCTA |
| UNC5B | GTCGGACACTGCCAACTATAC | CCGCCATTCACGTAGACGAT |
| EGFR | CCTGGTCTGGAAGTACGCAG | CTTCGCATGAAGAGGCCGAT |
| AXL | GTTTGGAGCTGTGATGGAAGGC | CGCTTCACTCAGGAAATCCTCC |
| MET | AGCGTCAACAGAGGGACCT | GCAGTGAACCTCCGACTGTATG |

**Table 1: Primers used for validation of differentially expressed genes by qPCR**
